# Gut Mycobiota of Three *Rhinopithecus* Species Provide New Insights into the Association Between Diet and Environment

**DOI:** 10.1111/1749-4877.12932

**Published:** 2024-12-17

**Authors:** Xuanyi Yang, Xiaochen Wang, Mingyi Zhang, Ying Shen, Yang Teng, Ming Li, Huijuan Pan

**Affiliations:** ^1^ School of Ecology and Nature Conservation Beijing Forestry University Beijing China; ^2^ CAS Key Laboratory of Animal Ecology and Conservation Biology, Institute of Zoology Chinese Academy of Sciences Beijing China; ^3^ College of Life Sciences University of Chinese Academy of Sciences Beijing China; ^4^ Center for Excellence in Animal Evolution and Genetics Chinese Academy of Sciences Kunming China

**Keywords:** feeding habit, fungi, gut mycobiota, living environment, *Rhinopithecus*

## Abstract

Gut mycobiota are part of the gut microbiome, typically derived from the host diet and living environment. In this study, we examined the gut mycobiota of three snub‐nosed monkeys: *Rhinopithecus roxellana*, *R. bieti*, and *R. strykeri* using next‐generation amplicon sequencing targeting the fungal internal transcribed spacer. The alpha diversity indexes of gut mycobiota in *R. bieti* were significantly higher than *R. roxellana and R. strykeri*, the beta diversity indicated that *R. roxellana* and *R. bieti* had more similar feeding habits. Core mycobiota demonstrated commonalities among the three species and potentially associated with feeding habits. Mycobiota displaying significant differences exhibited the respective characteristics of the host, likely associated with the hosts’ living environment. Among them, animal and plant pathogenic fungi and lichen parasites are potential threats to the survival of snub‐nosed monkeys for their pathogenicity to both monkeys and their food plants. Functionally, fungal trophic modes and functional guilds revealed a strong association between gut mycobiota and host diet. We found a higher abundance and more significant correlations with lichen parasitic fungi in *R. strykeri* than the other two species, indicating potential threats to their foods. Accordingly, this study revealed the basic structures of gut mycobiota of three wild *Rhinopithecus* species and highlighted the associations between gut mycobiota and their feeding habits and living environments. Furthermore, due to the close connection between fungi and the environment, animals could ingest fungi from their diet; thus, we speculate that gut mycobiota may serve a role in environmental monitoring for wildlife.

## Graphical abstract

This study revealed the basic structures of gut mycobiota of three wild *Rhinopithecus* species. The gut mycobiota of *Rhinopithecus* showed close connection with their feeding habits, and there were associations between gut mycobiota and the host's living environments.

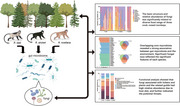

## 1 Introduction

The survival of nonhuman primates (NHPs) faces many challenges, especially fragmentation and population decline (Estrada et al. 2017). Some NHPs are on the brink of extinction due to habitat damage caused by deforestation and agricultural expansion (Fan, Fei, and Luo 2014; Mazumder 2014), including the Chinese colobine species *Rhinopithecus* spp. (Liu et al. 2015), which have been listed as endangered and critical endangered species by the IUCN (Geissmann, Momberg, and Whitten 2020; Long, Bleisch, and Richardson 2020; Yongcheng and Richardson 2021). Therefore, prioritizing the habitat quality of NHPs is crucial for the conservation of NHPs.

*Rhinopithecus* spp., also known as snub‐nosed monkeys, are primarily florivores living in temperate evergreen or deciduous broadleaf forests (>1500‐m altitude) (Chi et al. 2014; Kirkpatrick, Gu, and Zhou 1999; Long et al. 1994; Xiao et al. 2003). *R. roxellana*, for instance, consumes leaves in the summer and switches to barks and lichens in the winter (Fang et al. 2018). *R. bieti* feeds on lichens, various plant parts, and insects (Huang et al. 2017). *R. strykeri* feeds on buds and leaves in spring and summer, transitions to fruits and seeds from summer to fall, and relies on mature leaves in the winter (Yang et al. 2019). Snub‐nosed monkeys usually ingest different amounts of lichens (Ding and Zhao 2002; Fang et al. 2018; Xiang, Huo, and Xiao 2011; Yang 2019), which are fungal and algal symbionts (Song et al. 2022). Given their extensive active range and varied diet that includes fungi from their natural environment, snub‐nosed monkeys are ideal subjects for studying gut mycobiota.

With the development of high‐throughput sequencing technology, the gut microbiome has become an important tool for understanding the physiology, immune system, living environment, and lifestyle of wildlife (Pareek, Smoczynski, and Tretyn 2011; Turnbaugh et al. 2007; Zhang et al. 2024). Gut mycobiota are an integral component of the microbial composition in the gastrointestinal tract, but their role is often neglected. Fungi inhabit nearly all ecosystems on Earth and have vast diversity (Raspor and Zupan 2006). From lichen‐forming species that establish symbiotic relationships with algae (Song et al. 2022) to the myriad fungal inhabitants of soil (Ferreira et al. 2021) and freshwater habitats (Blackwell 2011), fungi play essential roles in nearly every environment. Endophytic fungi colonize the intercellular spaces of plant leaves and stems, forming intricate relationships with their hosts (Rodriguez et al. 2009). Fungi originating from food could colonize the intestinal tract after being ingested by the host. For instance, human consumption of fermented foods can lead to the colonization of the gut by species such as *Candida*, *Debaryomyces*, *Penicillium*, and *Scopulariopsis* (David et al. 2014). Herbivores and omnivores, which typically incorporate various plants into their diets, tend to have a more diverse gut mycobiota than insectivores or carnivores (Lavrinienko et al. 2021; Li et al. 2018). Moreover, lichens and plant‐related fungi although abundant in herbivores are seldom found in carnivores because of their different feeding habits (Jiang et al. 2020). Recent research has revealed that gut mycobiota in wild primates are often species‐specific, reflecting the nuanced dietary preferences of the host (Barelli et al. 2020). Notably, wild animals generally consume more fungi than their captive counterparts, likely because of their diverse range of food types (Siriyappagouder et al. 2018; B. Sun et al. 2021). Besides, gut mycobiota may reflect the condition of the host's environment. In various studies, the connection between the environment and gut mycobiota has been demonstrated. A study on Tibetan macaques observed that over 30% of amplicon sequence variants (ASVs) in the gut mycobiota of two wild groups in different geographic locations correlated with the local environmental samples, including plants and soil (B. H. Sun et al. 2021). Early‐life environmental factors could modify the gut mycobiota in children, indicating environmental fungi can colonize the human gut (Boutin et al. 2021). Moreover, *Aspergillus* species are more frequently found in environmental samples (soil, air, plant matter) than in gut samples but could survive the human physiological temperatures and colonize in the gut, suggesting they are primarily of environmental origin (O'Gorman and Fuller 2008; Mortensen et al. 2010). Therefore, investigating the relationship between gut mycobiota and the diet and living environment of the host may provide new insights into the conservation of snub‐nosed monkeys.

In NHPs, studies have mainly examined bacterial communities in the intestinal tract using 16S amplicon sequencing (Clayton et al. 2016; Gomez et al. 2015; Frankel et al. 2019), while mycobiota are less well‐studied compared with bacteria (Hufnagle and Noverr 2013). In this study, we would like to investigate the basic structure and predict the functions of gut mycobiota of the wild snub‐nosed monkeys. Furthermore, given the close relationship between gut mycobiota, diet, and environment, we hypothesized that gut mycobiota could reflect the diet and living environment of snub‐nosed monkeys and suggest whether the gut mycobiota can serve as a potential indicator for monitoring the quality of food and habitat of snub‐nosed monkeys. We sequenced the gut mycobiota of three free‐ranging *Rhinopithecus* species (*R. roxellana*, *R. bieti*, and *R. strykeri*) using ITS amplicon sequencing to understand the basic composition, structure, and function of the gut mycobiota of these species. We elucidated the relationship between the gut mycobiota, diet, and living environment of these species.

## 2 Material and Methods

### 2.1 Study Subjects and Sample Collection

We collected fecal samples from six sites in China, including samples of *R. roxellana* from Mianyang (*n* = 9) in Sichuan Province and Shennongjia (*n* = 14) in Hubei Province, samples of *R. bieti* from Tacheng (*n* = 10) and Mt. Lasha (*n* = 10) in Yunnan Province and Mangkang (*n* = 9) in Tibet, and fecal samples of *R. strykeri* from Mt. Gaoligong (*n* = 9) in Yunnan Province. In total, 61 fecal samples were obtained, 23 from *R. roxellana*, 29 from *R. bieti*, and 9 from *R. strykeri* (Table S1). All fecal samples were promptly collected post‐defecation under direct observation. The sampling time of feces is generally within 1–3 min after the defecation is observed, using sanitary disposable gloves to reduce the pollution. In addition, the majority of samples were obtained during winter, with feces deposited on snow, which typically has a lower fungal presence (Semenova et al. 2016; Mundra et al. 2016). Healthy individuals were identified based on the appearance of feces after defecation (Yang et al. 2022) and collected with sterile gloves. Detailed location, time, and individual information were recorded. Each cohort contained at least nine samples to ensure that the samples represented a group well (Knight et al. 2018). Samples of *R. roxellana* and *R. bieti* were snap‐frozen in liquid nitrogen, and samples of *R. strykeri* were stored in 95% ethanol (Vogtmann et al. 2017). The samples were transported on dry ice and stored frozen at −80°C until DNA extraction.

### 2.2 DNA Extraction and Sequencing

We extracted DNA from fecal samples using the cetyltrimethylammonium bromide method (Möller et al. 1992). During DNA extraction, the central portion of the fecal samples was selected to mitigate potential soil contamination. DNA concentration and purity were monitored on 1% agarose gel. DNA was diluted to 1 ng/µL using sterile water. PCR was performed using mixtures containing 15 µL of Phusion High‐Fidelity PCR Master Mix (New England Biolabs), 2 µM of forward and reverse primers, and 10 ng template DNA. The ITS regions are the universal DNA barcode marker for fungi (Schoch et al. 2012). We amplified the ITS1 subregion using primers ITS1‐1F‐F (5′‐CTTGGTCATTTAGAGGAAGTAA‐3′) and ITS1‐1F‐R (5′‐GCTGCGTTCTTCATCGATGC‐3′) (Nilsson, Anslan et al. 2019; Bokulich and Mills 2013). Sequencing libraries were generated using the TruSeq DNA PCR‐Free Sample Preparation Kit (Illumina, Inc., San Diego, USA) following the manufacturer's recommendations, and 250‐bp paired‐end reads were performed on the Illumina NovaSeq platform (Illumina, Inc., San Diego, USA). All PCR reactions were conducted using 15 µL of Phusion High‐Fidelity PCR Master Mix (New England Biolabs), 2 µM of forward and reverse primers, and approximately 10 ng of template DNA. The thermal cycling procedure involved an initial denaturation at 98°C for 1 min, followed by 30 cycles of 10 s at 98°C for denaturation, 30 s at 50°C for annealing, and 30 s at 72°C for elongation. The procedure concluded with a final extension at 72°C for 5 min. The PCR products were mixed in equidensity ratios, mixed with the same volume of 1X TAE buffer, and subjected to electrophoresis on a 2% agarose gel for detection. The mixture was purified using a Qiagen Gel Extraction Kit (Qiagen, Germany).

### 2.3 Bioinformatics and Identification of Fungal Taxa

Raw fungal ITS gene sequence data in FASTQ format were imported in QIIME 2 (Bolyen et al. 2019) using the qiime tools import program. Then, q2‐demux was used to demultiplex the sequences. Data were denoised using the DADA2 plugin; sequences were truncated to remove low‐quality regions, primers, and chimeras (Callahan et al. 2016). Then, the feature table of the ASV was obtained. We used VSEARCH (Rognes et al. 2016) to assign the taxonomy of ASV sequences, and the fungal feature table was generated using the UNITE v.8 database (https://unite.ut.ee) (Nilsson, Larsson et al. 2019; Table S2). We screened the core taxa of fungi at the family and genus levels to reduce the impact of small amounts of fungi incidentally carried in the environment around feces and rarely consumed food. The core mycobiota at the family and genus levels were defined as ASVs detected in at least 90% of samples, with average relative abundance >1% (Neu, Allen, and Roy 2021).

### 2.4 Fungal Diversity and Community Analysis

The microbial communities of the fecal samples were analyzed using QIIME 2 (Bolyen et al. 2019). We used relative abundance at the genus level to calculate alpha and beta diversity indexes. Alpha diversity indices, including the Chao 1 richness estimator and Simpson's diversity index, were calculated to reflect microbial diversity between the three species. One‐way ANOVA and Bonferroni's multiple comparison tests were performed to measure statistical significance within groups, and *p*‐values were adjusted by Bonferroni's correction. The Bray–Curtis index was used to construct the distance matrix. Differences between fungal communities were determined and visualized by principal co‐ordinates analysis (PCoA) to reflect the structural variation of fungal communities (Anderson and Willis 2003). The group distance results were tested using permutational multivariate ANOVA (PERMANOVA) to ensure normal contribution (Anderson and Walsh 2013). Linear discriminant analysis effect size (LEfSe) (https://huttenhower.sph.harvard.edu/galaxy/ (accessed on July 15, 2022)) was used to detect the taxonomy with significant differences between the three species. Taxa with LDA scores >4.0 were retained. Correlation networks were constructed to exhibit the connections between significantly different taxa at the genus level and fugal functional guilds, calculated by Spearman's correlation coefficient and partial Mantel test. Functions of fungal communities (trophic modes and functional guilds) were predicted using the FUNGuild database (Nguyen et al. 2016). The FUNGuild database sorted the fungal functions by the way they receive nutrients. For instance, saprotrophic fungi decompose organic matter and mobilize the nutrient and carbon cycle (Van der Wal et al. 2013). Symbiotrophic fungi live in plant roots and can help plants absorb nutrients (Kramer et al. 2012).

Assignments with a confidence score of “probable” or “highly probable” were selected; functions with a lower confidence score of “possible” were not selected. Kruskal–Wallis ANOVA with Dunn's multiple comparison test was used to compare the relative abundance of functional guilds within the three species. ANOVA, Bonferroni's multiple comparison test, and visualization of boxplots were performed using GraphPad Prism v.9.0.0 (GraphPad, San Diego, CA, USA). In all analyses, *p* < 0.05 was considered statistically significant. Linear regression and both alpha and beta diversity indices were calculated through R (Ihaka and Gentleman 1996), and their visualization was implemented through the ggplot2.

## 3 Results

### 3.1 Basic Structure and Diversity of Mycobiota Between the Three Species

Bioinformatic processing was used to obtain 19 799 high‐quality sequences from the feces of 23 *R. roxellana*, 29 *R. bieti*, and 9 *R. strykeri* samples (Table S1). Filtering and taxonomic assignment identified 13 phyla, 53 classes, 158 orders, 395 families, 1090 genera, and 2053 fungal species (Table S2).

In total, 10 152 fungal ASVs were obtained in all samples (*R. bieti*: 6265, *R. roxellana*: 3965, and *R. strykeri*: 1441). In total, 268 ASVs were shared by the three species, suggesting that the composition of gut mycobiota of the three snub‐nosed monkeys share similarities. In total, 967, 500, and 320 ASVs were shared between *R. roxellana* and *R. bieti*, *R. bieti* and *R. strykeri*, and *R. roxellana* and *R. strykeri*, respectively, indicating the most similarity between *R. roxellana* and *R. bieti* and greatest difference between *R. roxellana* and *R. strykeri* (Figure S1).

**FIGURE 1:**
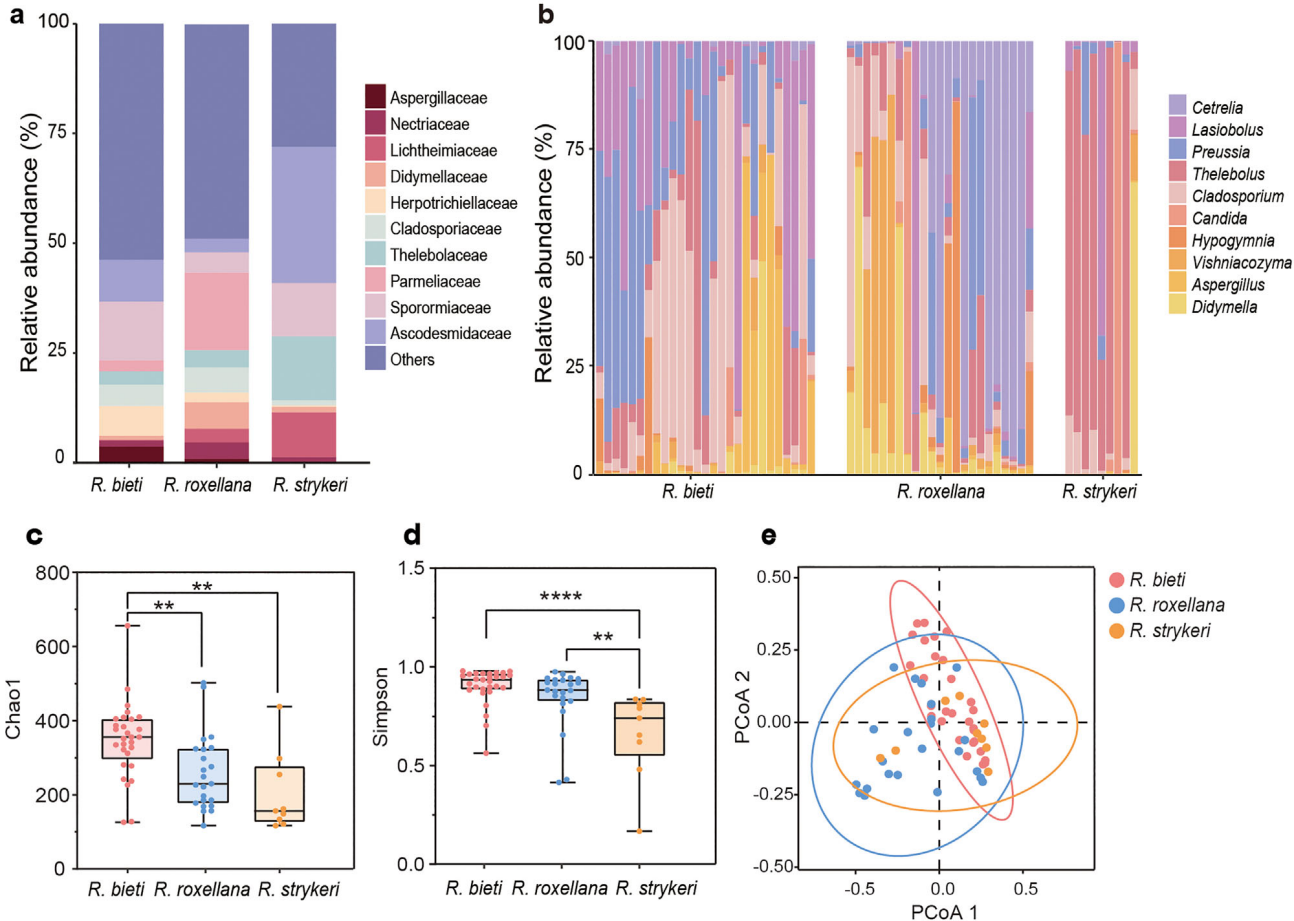
Relative abundances of the fungal taxonomy and comparison of alpha and beta diversity in the three *Rhinopithecus* species. (a) The distribution of the most abundant fungi at the genus level in each sample. Only the top 10 taxa are shown. (b) The distribution of the most abundant fungi at the genus level in each species. (c) Chao 1 index representing the total ASV number in each species. (d) Simpson's diversity index representing ASV richness in each species (one‐way ANOVA test with Bonferroni's multiple comparison test, **p < 0.01, ****p < 0.0001). (e) Bray–Curtis distance of PCoA representing the calculated distance between the three species (permutational multivariate analysis of variance, PERMANOVA, *p* = 0.001).

We analyzed the relative abundance of fungal taxa at different taxonomic levels in all samples. At the phylum level, the most abundant phyla were Ascomycota (mean ± SD, *R. bieti*: 69.3% ± 13.4%; *R. roxellana*: 66.5% ± 25.5%; *R. strykeri*: 82.6% ± 25.0%) and Basidiomycota (*R. bieti*: 12.3% ± 10.3%; *R. roxellana*: 18.0% ± 19.5%; *R. strykeri*: 11.2% ± 23.1%; Figure S2). At the family level, Ascodesmidaceae, Sporormiaceae, Parmeliaceae, Thelebolaceae, and Cladosporiaceae were ranked as the five most abundant fungi (Figure S3 and Table S2). Among the three species, the five most dominant mycobiota at the genus level were *Thelebolus*, *Candida*, *Lasiobolus*, *Preussia*, and *Cladosporium* (Figure 1a,b; Table S2).

Alpha diversity was measured using the Chao 1 and Simpson indexes. The Chao 1 index of *R. bieti* was significantly higher than that of *R. roxellana* and *R. strykeri* (*p* < 0.01 and *p* < 0.005); the difference between *R. roxellana* and *R. strykeri* was insignificant (Figure 1c; Table S3). *R. bieti* and *R. roxellana* had significantly higher values of the Simpson index than *R. strykeri* (*p* < 0.0001) (Figure 1d; Table S3). *R. bieti* had the highest alpha diversity compared with the other species. Beta diversity of PCoA was examined based on Bray–Curtis dissimilarity, indicating that the fungal communities within the species clustered closely and significantly differed between the cohorts (PERMANOVA, *p* < 0.005). The sample points with close distances suggested similarity in microbial diversity and composition of the three species; besides, *R. strykeri* exhibited higher similarity with *R. roxellana*; *R. roxellana* and *R. bieti* had greater differences (Figure 1e).

### 3.2 Relationship Between Fungal Taxa and Diet of the Three Snub‐Nosed Monkey Species

**FIGURE 2:**
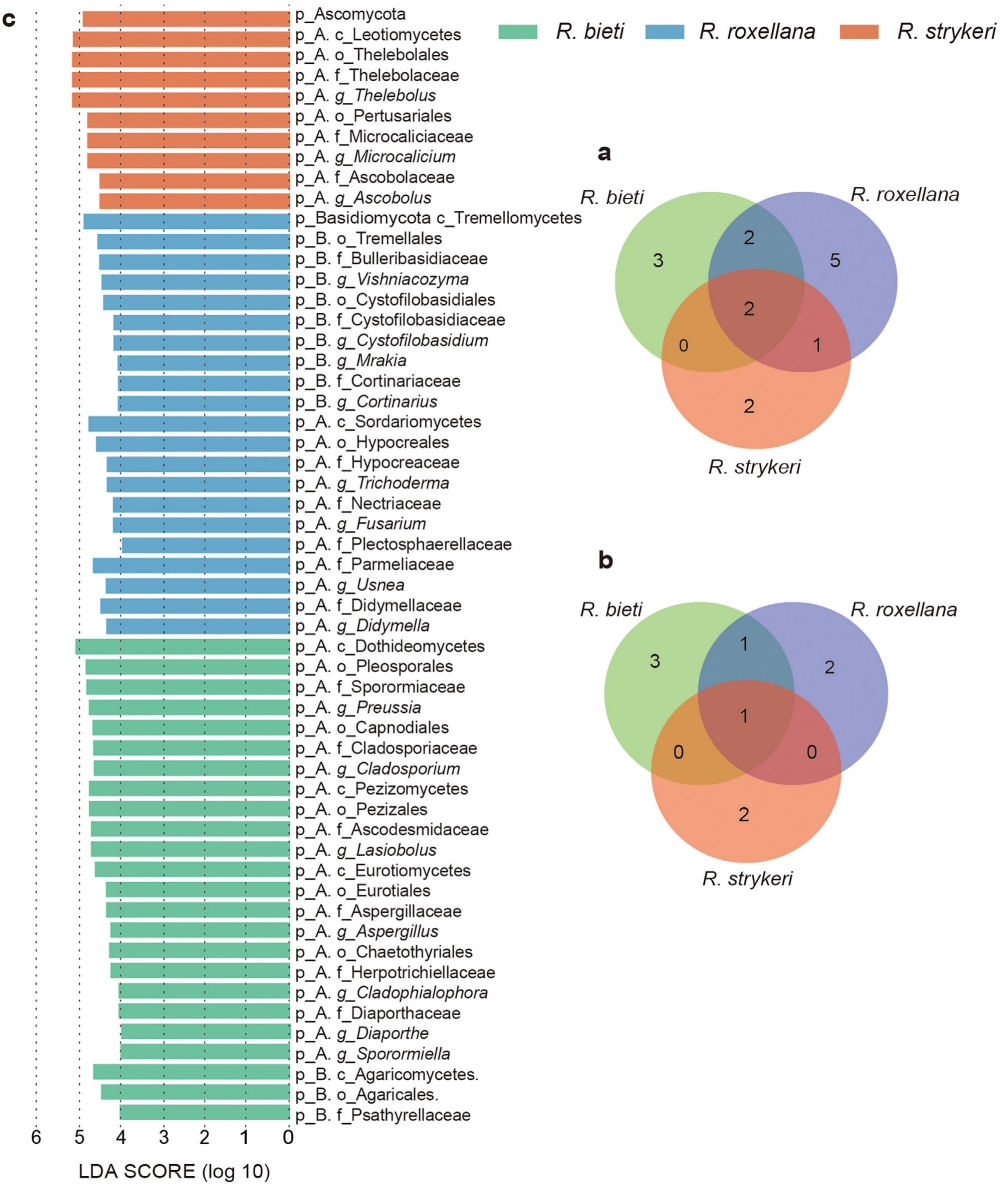
The core and significant fungal taxonomy in all samples. (a) Unique and shared core mycobiota in the samples of the three species at the family level. (b) Unique and shared core mycobiota in the samples of the three species at the genus level. (c) Linear discriminant analysis effect size (LEfSe) to identify fungal taxa with significant differences between the species from phylum to genus level (LDA > 4.0, *p* < 0.05).

At the family level, 10, 7, and 5 core fungal taxa were identified in *R. roxellana*, *R. bieti*, and *R. strykeri*, respectively (Figure 2a; Table S4). The two most predominant families in each species were Parmeliaceae (18.8%) and Didymellaceae (6.1%) in *R. roxellana*, Sporormiaceae (11.5%) and Ascodesmidaceae (4.8%) in *R. bieti*, and Thelebolaceae (13.8%) and Sporormiaceae (9.8%) in *R. strykeri*. At the genus level, 4, 5, and 3 core taxa were detected in *R. roxellana*, *R. bieti*, and *R. strykeri*, respectively (Figure 2b; Table S4), of which the most predominant genera were *Vishniacozyma* (4.4%) and *Thelebolus* (3.2%) in *R. roxellana*; *Lasiobolus* (8.7%) and *Preussia* (6.9%) in *R. bieti*; and *Thelebolus* (13.8%) and *Microcalicium* (5.7%) in *R. strykeri*. Sporormiaceae and Thelebolaceae were overlapping families between *R. bieti* and *R. strykeri*, and Thelebolaceae was shared between all species. Notably, overlapping core mycobiota were detected between the three species. At the genus level, *Cladosporium* and *Thelebolus* overlapped in *R. bieti* and *R. roxellana*. *Thelebolus* was overrepresented in all species. *Cladosporium* was overrepresented in *R. bieti*, with an occurrence rate of 100%. Overlapping core mycobiota suggest the associations between gut mycobiota and the diet. In addition, the proportion of lichen intake in the diet of three snub‐nosed monkeys in our sampling sites and the relative abundance of fungi family, Parmeliaceae and Ramalinaceae, which are also the main lichen family consumed by three snub‐nosed monkeys, are corroborated by a positive association (Table S7; Figure S5).

### 3.3 Significant Fungal Taxa Reflect the Characteristics of Each Species

The histogram result showed 55 distinctly abundant fungal communities across all three species, of which there were 21 in *R. roxellana*, 24 in *R. bieti*, and 10 in *R. strykeri* (Figure 2c; Table S5). According to taxonomic classification, we divided the 55 significant taxa of different classification levels into several main families or genera. In *R. roxellana*, the significant taxa included the families Bulleribasidiaceae, Didymellaceae, and Plectosphaerellaceae and the genera *Vishniacozyma*, *Ascobolus*, *Usnea*, *Cortinarius*, *Fusarium*, *Trichoderma*, *Cystofilobasidium*, and *Mrakia*. In *R. bieti*, the significant taxa included *Lasiobolus*, *Cladosporium*, *Aspergillus*, *Diaporthe*, *Sporormiella*, *Cladophialophora*, and *Preussia*. In *R. strykeri*, the significant taxa were the genera *Ascobolus*, *Thelebolus*, and *Microcalicium*.

### 3.4 Main Functions of Mycobiota and Correlations With Significant Genus

**FIGURE 3:**
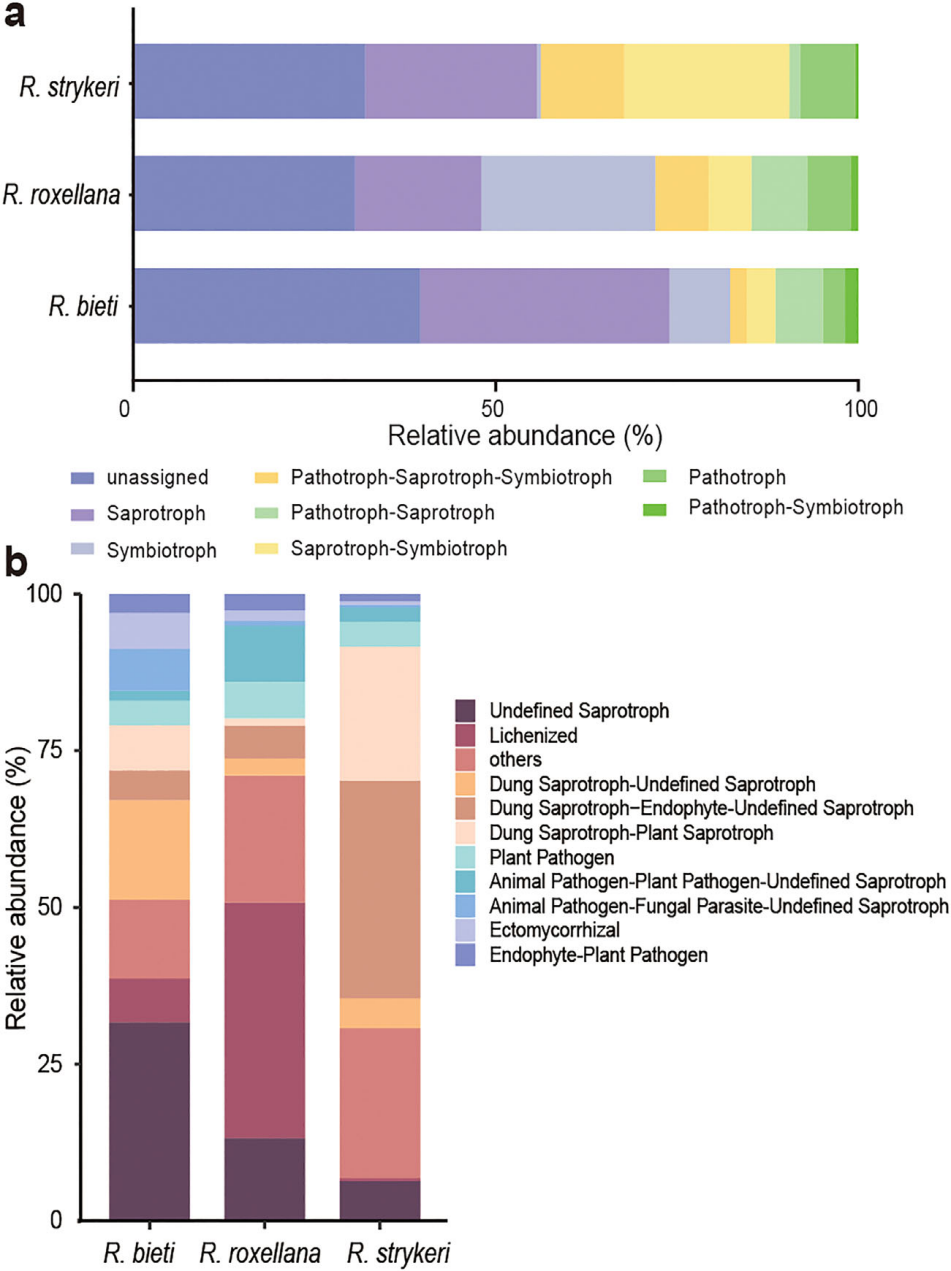
Assessing the functions of mycobiota. (a) Variation of fungal trophic modes. Each column shows the relative abundance of the trophic modes of each species. (b) Comparison of functional guilds. Functional guilds with significant differences (Dunn's multiple comparison test, *p* < 0.05) in all samples; relative abundance of top 10 taxa are shown.

The FUNGuild database was used to identify the trophic modes and functional guilds of the fungi (Nguyen et al. 2016). The relative abundance of trophic modes showed that Saprotroph (35.1%), Symbiotroph (26.4%), and Saprotroph–Symbiotroph (13.1%) were the most abundant trophic modes among the three species (Figure 3a; Table S6). In *R. roxellana*, Symbiotroph (24.0%) was the most dominant trophic mode, followed by Saprotroph (17.4%). In *R. bieti*, Saprotroph (34.4%) was the most dominant trophic mode, followed by Symbiotroph (8.3%). In *R. strykeri*, Saprotroph (23.7%) was the most abundant, and Saprotroph–Symbiotroph (22.8%) ranked second (Figure 3a; Table S6).

**FIGURE 4:**
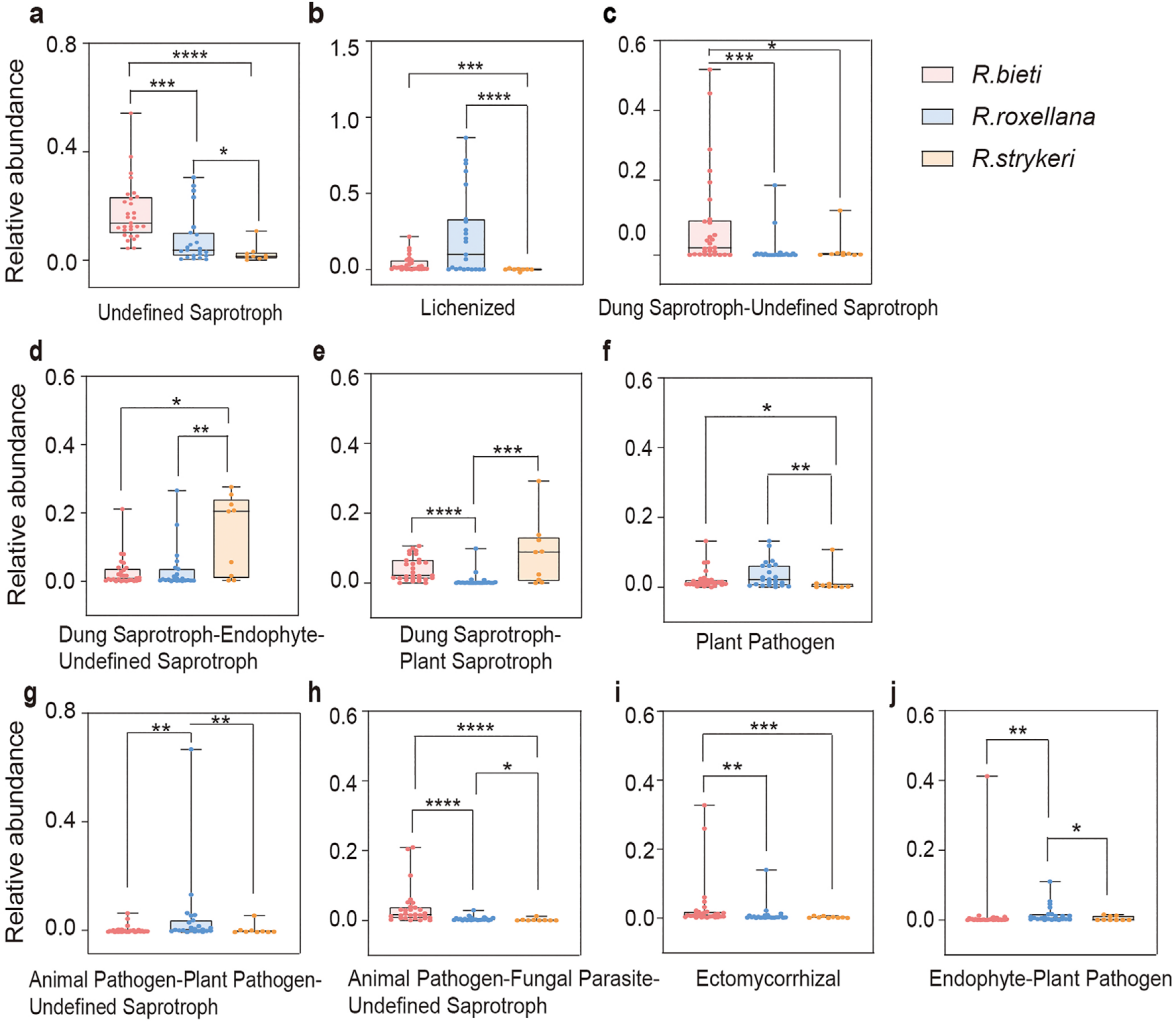
The most abundant guilds of all samples. (a–j) Boxplot exhibiting relative abundance of the 10 most abundant guild of all samples, differences within the group were calculated (*p < 0.05, **p < 0.01, ***p < 0.001, ****p < 0.0001).

Functional guilds focus on trophic strategies and help distill complex taxonomical communities into more manageable units (Nguyen et al. 2016). In all cohorts, the most abundant known guilds were Undefined Saprotroph (11.7%), Lichenized (10.4%), Dung Saprotroph–Undefined Saprotroph (5.1%), Dung Saprotroph–Endophyte–Undefined Saprotroph (4.5%), and Dung Saprotroph–Plant Saprotroph (3.4%) (Figure 3b; Table S6). Within each cohort, saprotrophic fungi (Undefined Saprotroph, 17.5%; Dung Saprotroph–Undefined Saprotroph, 8.8%; Dung Saprotroph–Plant Saprotroph, 4.0%) were significantly more abundant in *R. bieti*, whereas the relative abundance of lichenized fungi (Lichenized, 22.7%) and animal pathogens (Animal Pathogen–Plant Pathogen–Undefined Saprotroph, 5.4%) was significantly higher in *R. roxellana*. In addition, *R. strykeri* had a higher relative abundance of saprotrophic fungi (Dung Saprotroph–Endophyte–Undefined Saprotroph, 13.8%; Dung Saprotroph–Plant Saprotroph, 8.5%), followed by lichen parasite fungi (Lichen Parasite, 5.7%; Figure 4; Table S6). Thus, the dominant functions of the three species were dung saprotrophs, lichenized fungi, and endophytes. Notably, *R. roxellana* had a higher abundance of animal pathogens than *R. strykeri* and *R. bieti*, indicating higher disease risk (Lorenz, Bender, and Fink 2004; Sartor and Wu 2017)

**FIGURE 5:**
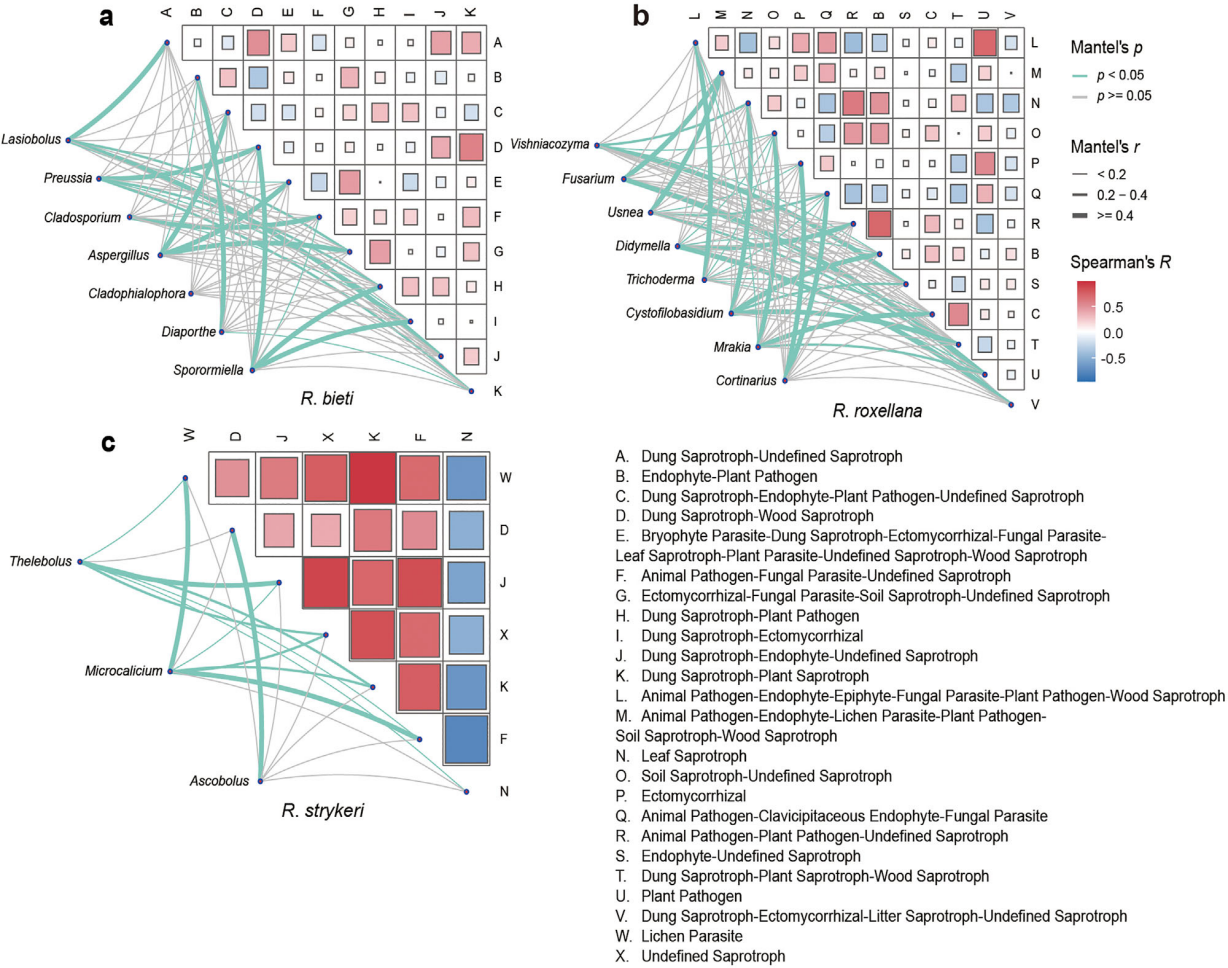
Correlations of functional guilds and significant fungal taxa. (a–c) Correlation heatmap of functional guilds and significant genus (LDA 4.0) of each species. Significant fungal communities correlated with guilds by Mantel's test. Light green lines represent positive correlations and gray lines represent negative correlations. The width of the line corresponds to Mantel's *r* statistic; the color gradient represents Spearman's correlation coefficient.

In all samples, significant genera were positively correlated with saprotrophs, animal and plant pathogens, lichen parasites, ectomycorrhiza, and endophytes (Figure 5). In *R. bieti*, significant mycobiota were predominantly associated with saprotrophs. In *R. roxellana*, significant mycobiota had more positive correlations with animal pathogens than the other species. Uniquely, *R. strykeri* showed a positive association with lichen parasite fungi. In addition, analysis of the correlations between functional guilds and core mycobiota at the genus level showed more positive correlations with dung saprotrophs in *R. bieti* than with *R. roxellana* and *R. strykeri* (Figure S4). Other correlations mainly concentrated on lichen parasites, animal pathogens, plant pathogens, and saprotrophs.

## 4 Discussion

Our study revealed the basic structure and characteristics of the gut mycobiota of three species of *Rhinopithecus* and compared the composition and function of their gut mycobiota. The results indicated that the gut mycobiota of the three *Rhinopithecus* species are closely related to their diet. Due to the close relationship between fungi and the environment, the gut mycobiota reflect not only the feeding habits but also indicate the living environment of wild animals to some extent.

Basic structure exhibited the composition of gut mycobiota of three snub‐nosed monkeys. Ascomycota and Basidiomycota were the most abundant phyla, which constitute the primary groups of terrestrial fungi (Redecker, Kodner, and Graham 2000) and have been identified as the predominant fungal phyla in several mammalian species, including humans (Borges et al. 2018; Chen, Chen, and Wang 2023; Gouba and Drancourt 2015; Li et al. 2021; Lv et al. 2023; Yang et al. 2018). Most fungi with high relative abundance were associated with the natural environment and plants. For instance, the family Didymellaceae includes saprophytic, endophytic, and pathogenic species, with some being widespread plant pathogens (Khodaei et al. 2018). The family Parmeliaceae is involved in lichen formation (Crespo et al. 2010). Similarly, genera such as *Trichoderma*, *Cladosporium*, *Diaporthe*, and *Aspergillus* are common plant pathogenic fungi (Frisvad and Larsen 2015; Gomes et al. 2013; Vinale et al. 2008; Zhang et al. 2024). *Preussia* and *Sporormiella* are dung‐inhabiting fungi (Mapperson et al. 2014; Etienne et al. 2013). Among these, only *Candida* was a common pathogenic fungus associated with gastrointestinal diseases (Mukherjee et al. 2015; Sokol et al. 2017). Alpha diversity indexes of *R. bieti* were significantly higher than those of *R. strykeri* and *R. roxellana*, indicating a richer variety of food in wild *R. bieti* (Ding and Zhao 2002; Yang 2019). Beta diversity analysis revealed more similarities between the fungal communities of *R. bieti* and *R. strykeri*. This might be due to the higher proportion of lichen intake in *R. roxellana* and *R. bieti* compared to *R. strykeri*. Indeed, the higher number of fungal ASVs in *R. bieti* and *R. roxellana* than in *R. strykeri* also suggests more abundant fungi in their diet.

Defining the core mycobiota could more specifically reflect the information of the host and reduce the impact of exotic factors (Neu, Allen, and Roy 2021). We screened the core mycobiota at both family and genus levels to identify representative mycobiota for each species. We found that the general consistency of core mycobiota between the three species was related to their diet and could discern shared characteristics of gut mycobiota between the three species by examining overlapping core fungi. For instance, Parmeliaceae, the most common lichen‐forming fungi (Crespo et al. 2010), emerged as a shared core fungal family for *R. roxellana* and *R. bieti*. This aligns with previous studies showing that lichens belonging to Parmeliaceae are primarily consumed by these two species (Grueter et al. 2009; Li, Yang, and Yang 2011; Tie 2013; Yang 2019). Furthermore, research on the feeding habits of snub‐nosed monkeys has demonstrated varying proportions of lichen consumption: 50.6%–82.5% for *R. bieti*, 2.8%–38.4% for *R. roxellana*, and 2.5% for *R. strykeri* (Ding and Zhao 2002; Xiang, Huo, and Xiao 2011; Yang et al. 2019; Yang 2019; Fang et al. 2018). These findings corroborate our observations of a higher relative abundance of Parmeliaceae in *R. bieti* and *R. roxellana* compared to *R. strykeri*, suggesting a strong correlation between gut mycobiota and dietary fungal content in snub‐nosed monkeys. Additionally, the common core family Sporormiaceae, which comprises mostly coprophilous fungi (Kruys and Wedin 2009), and Thelebolaceae, which includes several coprophilous fungi (Landvik, Kristiansen, and Schumacher 1998), were identified across all three species. This is consistent with studies reporting the presence of coprophilous fungi in herbivore feces (Caretta et al. 1998), likely reflecting the herbivorous diet of snub‐nosed monkeys. Moreover, the common core genus *Thelebolus* has been identified in the feces of wild long‐tailed gorals (Park et al. 2023) and other ruminants, such as cows and yaks (Zhu et al. 2023; Tong et al. 2023; Yang et al. 2023), which might be related to the special foregut fermentation digestion system of snub‐nosed monkeys (Zhou et al. 2014; Liu et al. 2022).

Through the screening of significantly different fungi, we found these significant fungal taxa in the three species of snub‐nosed monkeys might reflect certain characteristics of the host to some extent. Parmeliaceae, *Usnea*, and *Vishniacozyma* are lichen‐forming fungi (Lindgren et al. 2015; Peintner, Moncalvo, and Vilgalys 2004; Schmitt et al. 2005); these are the significant fungi of *R. roxellana*, which could be related to the lichen intake in their diet. *Fusarium* is also a significant fungi in *R. roxellana*. It is a widespread and important plant pathogenic fungi, renowned for its virulence and infectiousness to crops, with the ability to spread through infected plants (Ma et al. 2014). Meanwhile, it is also a common pathogen associated with animal and human diseases, such as leukemia, which can be transmitted by consuming infected plants (Nelson, Dignani, and Anaissie 1994). *Aspergillus* and *Cladophialophora* are the significant fungi in *R. bieti*; these two genera are also common pathogenic fungi. *Aspergillus* could produce mycotoxin aflatoxin, which is highly carcinogenic and hepatotoxic (Bennett and Klich 2003), and lead to a variety of lung diseases (Brown et al. 2012). *Cladophialophora* are yeast‐like fungi, including species that cause cerebral and skin infections (Badali et al. 2008). Notably, the presence of *Aspergillus* and *Fusarium* in the gut mycobiota of snub‐nosed monkeys aligns with previous findings in Sumatran orangutans (*Pongo abelii*), Tibetan macaques (*Macaca thibetana*), and lemurs (*Indri indri*) (Safika et al. 2023; Sun et al. 2018; Borruso et al. 2021). This consistency suggests that these genera may be potential common pathogens for primates.

Saprotrophs are crucial to decomposition and nutrient cycling, possessing the ability to break down lignin and cellulose found in leaves and wood, as well as to degrade chemical pollutants within the soil (Ceci et al. 2019). This suggests that fungi originating naturally from environments such as soil and leaf litter may potentially colonize the gut of snub‐nosed monkeys. Except for the high relative abundance of lichenized guild, which is associated with their lichen intake, the presence of plant‐related fungi, such as mycorrhizae and endophytes, which generally inhabit different parts of plants (Miller and Jastrow 2000; Stone et al. 2004), indicates the diversity of plant parts consumed by snub‐nosed monkeys. Consistently, a study on a highly specialized frugivorous bat (*Ectophylla alba*) found similarities between the functions of bat gut mycobiota and their food source, *Ficus colubrinae* (Chaverri and Chaverri 2022), highlighting the close relationship between host gut mycobiota and consumed food. Our results revealed a higher abundance and significant correlation with lichen parasites in *R. strykeri*, indicating the overgrowth of lichen parasites. Lichen parasites are harmful to lichen growth and can cause the death of the lichens (Hawksworth 1983; Merinero and Gauslaa 2018); it is a non‐negligible threat to *R. strykeri* survival. This phenomenon might result from the extensive loss of their habitat (Ren et al. 2017). Furthermore, the presence of animal pathogens at varying abundance levels across the samples suggests that the threat of fungi to the health of wild animals should not be ignored (Fisher et al. 2020).

Overall, gut mycobiota of snub‐nosed monkeys are highly related to their diet and showed potential connections with the living environment (Hallen‐Adams and Suhr 2017). Thus, we believe that gut mycobiota can be used as an indicator to simultaneously monitor the living environment and dietary status of wild animals (Lavrinienko et al. 2021). However, fully leveraging this potential requires a comprehensive understanding of environmental fungi and plant‐associated fungi. Conducting research on large wild populations to identify fungal colonizers presents significant challenges, primarily due to the limitations of mycological classification and the difficulties associated with sampling in natural habitats. Moreover, the complex interactions between bacteria and fungi represent another crucial aspect of microbiome research. Bacteria could promote fungal colonization in the host gut (Fan et al. 2015; Xu et al. 2008), while certain bacterial metabolites can inhibit fungal growth, potentially restraining fungal infections in the host (Noverr et al. 2004; Cottier et al. 2015). The interplay between bacteria and fungi plays a pivotal role in regulating the host's immune system. Given the significance of these microbial interactions and their potential impact on host health, further research in this area is necessary.

## 5 Conclusions

In summary, our findings demonstrated the composition and basic function of gut fungi of wild *Rhinopithecus* and described the source of gut fungi and their connection with their host. First, our study revealed the basic structures and diversity of the gut mycobiota in three free‐ranging *Rhinopithecus* species, indicating unique fungi within each cohort and commonly shared fungi between the three species. Second, the gut mycobiota of *Rhinopithecus* were closely connected with their feeding habits, and the abundance of fungi was significantly related to their diverse food range. Finally, we suggest that the presence of animal pathogens may be a potential fungal disease threat to wild *Rhinopithecus*. Our study provides a database of gut mycobiota for wild *Rhinopithecus*, which will be useful in evaluating the current status of *Rhinopithecus* survival and be a tool to monitor other wild NHPs in changing environments.

## Conflicts of Interest

The authors declare no conflicts of interest.

## Supporting information




**Table S1** Metadata of the samples, including sampleID, sample name, host, locations, longitude and latitude, and altitude.

**Table S2** The most anundant genus ranked top10.

**Table S3** Alpha diversity of Simpson index.

**Table S4** Core mycobiota of each species at genus level.

**Table S5** The result of Linear discriminant analysis Effect Size (LEfSe).

**Table S6** Relative abundance of significant functional guild.

**Table S7** The type of lichens consumed by *R. bieti, R. roxellana*, and *R. strykeri*, and average relative abundance of fungal family that consist the lichen in this study.
**Figure S1** Unique and shared fungal ASVs in the samples of three species.
**Figure S2** The distribution of the most abundant fungi at the phylum level in each species.
**Figure S3** The distribution of the most abundant fungi at the family level in each species.
**Figure S4** Correlation heatmap of functional guilds and core fungal community of each group.
**Figure S5** Correlation between the proportion of lichen intake in diet and relative abundance of fungi family.

## Data Availability

The data that support the findings of this study are openly available in NCBI Sequence Read Archive at https://www.ncbi.nlm.nih.gov/, reference number: PRJNA1080018.
