# Supplementary material for: Gut Mycobiota of Three Rhinopithecus Species Provide New Insights into the Association Between Diet and Environment
Source: Integr Zool. 2024 Dec 17;20(5):936–47. doi: 10.1111/1749-4877.12932 (PMC12463750; doi:10.1111/1749-4877.12932)
Supplement: Supplementary file 7 — Table S7 The type of lichens consumed by R. bieti, R. roxellana, and R. strykeri, and average relative abundance of fungal family that consist the lichen in this study. Figure S1 Unique and shared fungal ASVs in the samples of three species. Figure S2 The distribution of the most abundant fungi at the phylum level in each species. Figure S3 The distribution of the most abundant fungi at the family level in each species. Figure S4 Correlation heatmap of functional guilds and core fungal community of each group. Figure S5 Correlation between the proportion of lichen intake in diet and relative abundance of fungi family. [file INZ2-20-936-s002.doc]

**Supplementary Materials**

**Supplementary Tables**

**Supplementary Table S7.** The type of lichens consumed by *R.bieti*, *R.roxellana* and *R.strykeri*, and average relative abundance of fungal family that consist the lichen in this study.

| **Species** | **Latin Name** | **Family** | **Average Relative Abundance** |
| --- | --- | --- | --- |
| *Rhinopithecus bieti* | *Usnea longissima* | Parmeliaceae | 0.028171913 |
|  | *Bryoria confusa* |  |  |
|  | *Usnea florida* |  |  |
| *Rhinopithecus roxellana* | *Usnea longissima* | Parmeliaceae | 0.187824945 |
|  | *Usnea montis-fuji* |  |  |
|  | *Usnea aciculifera* |  |  |
|  | *Usnea luridorufa* |  |  |
| *Rhinopithecus strykeri* | *Dolichousnea longissima* | Parmeliaceae | 0.000747805 |
|  | *Nephromopsis nephromoides* |  |  |
|  | *Nephromopsis ornata* |  |  |
|  | *Nephromopsis pallescens* |  |  |
|  | *Nephromopsis stracheyi* |  |  |
|  | *Usnea florida* |  |  |
|  | *Usnea cavernosa* |  |  |
|  | *Usnea comosa* |  |  |
|  | *Usnea bismolliuscula* |  |  |
|  | *Usnea himantodes* |  |  |
|  | *Usnea sp.* |  |  |
|  | *Ramalina conduplicans* | Ramalinaceae | 5.52044E-05 |
|  | *Ramalina roesleri* |  |  |
|  | *Ramalina sinensis* |  |  |

**Supplementary Figures**


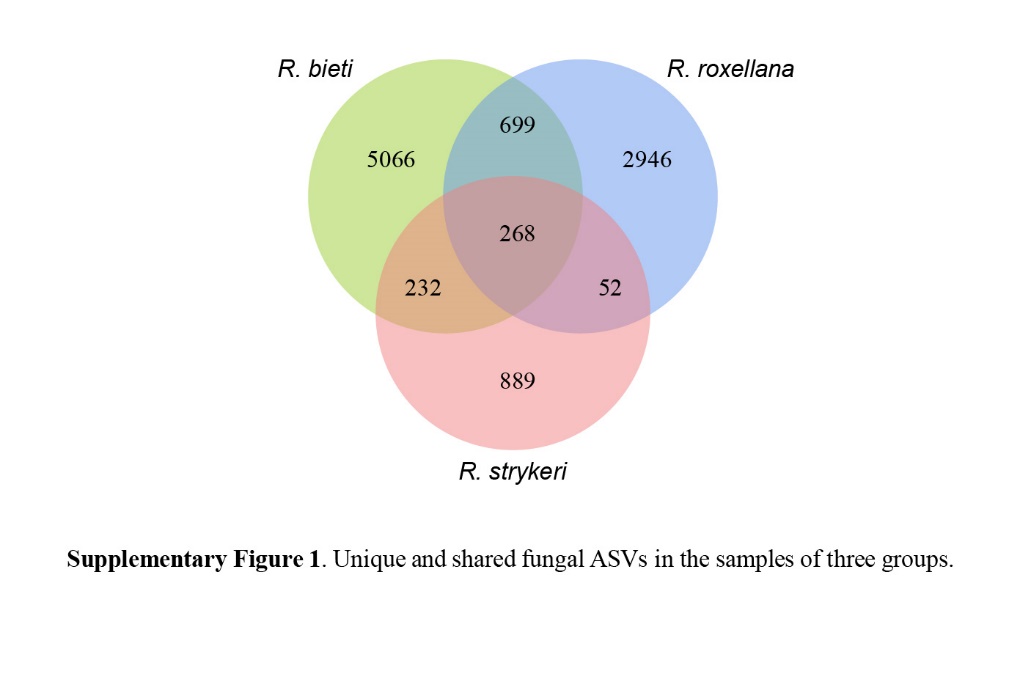


**Fig S1**. Unique and shared fungal ASVs in the samples of three species.


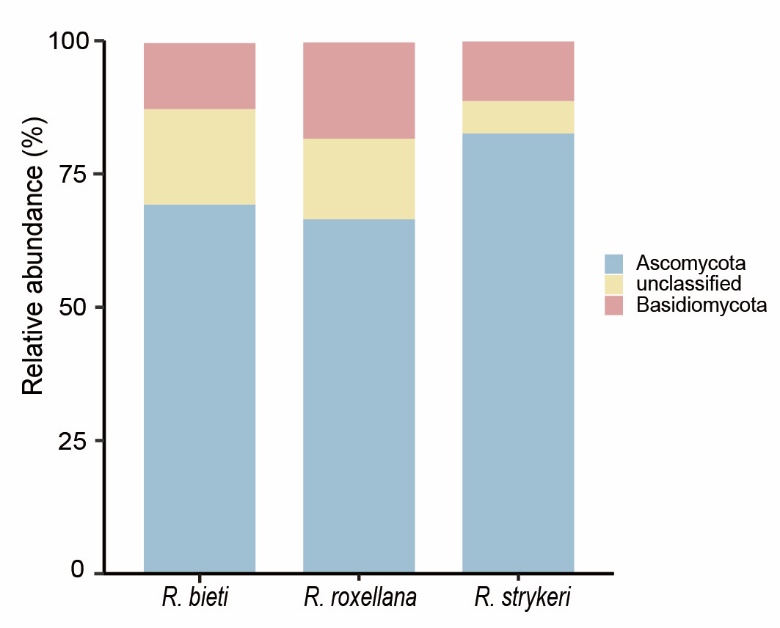


**Fig S2**. The distribution of the most abundant fungi at the phylum level in each species.


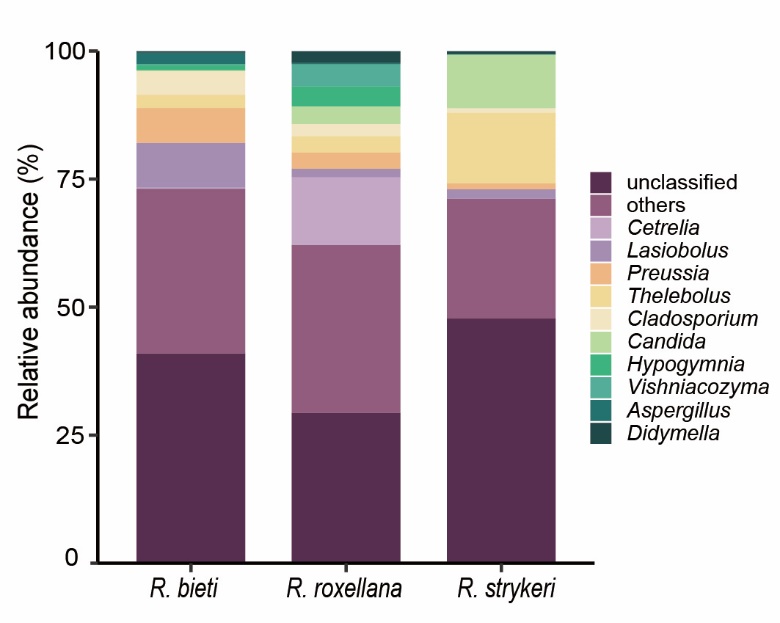


**Fig S3**. The distribution of the most abundant fungi at the family level in each species.


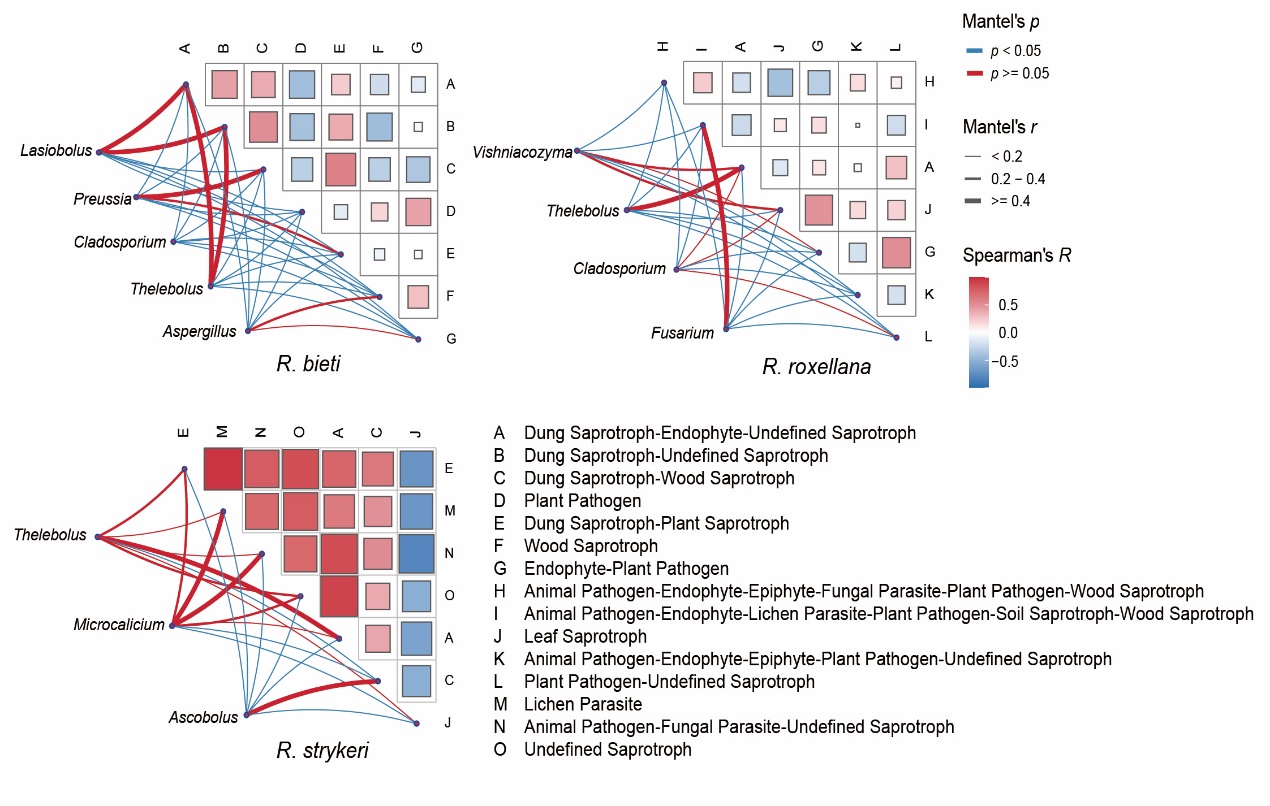


**Fig S4**. Correlation heatmap of functional guilds and core fungal community of each group. The core fungal communities were correlated with guilds by Mantel test. Red lines represent positive correlations and blue lines represent negative correlations, the width of the line corresponds to the Mantel’s statistic, color gradient noted the Pearson’s correlation coefficient.

**
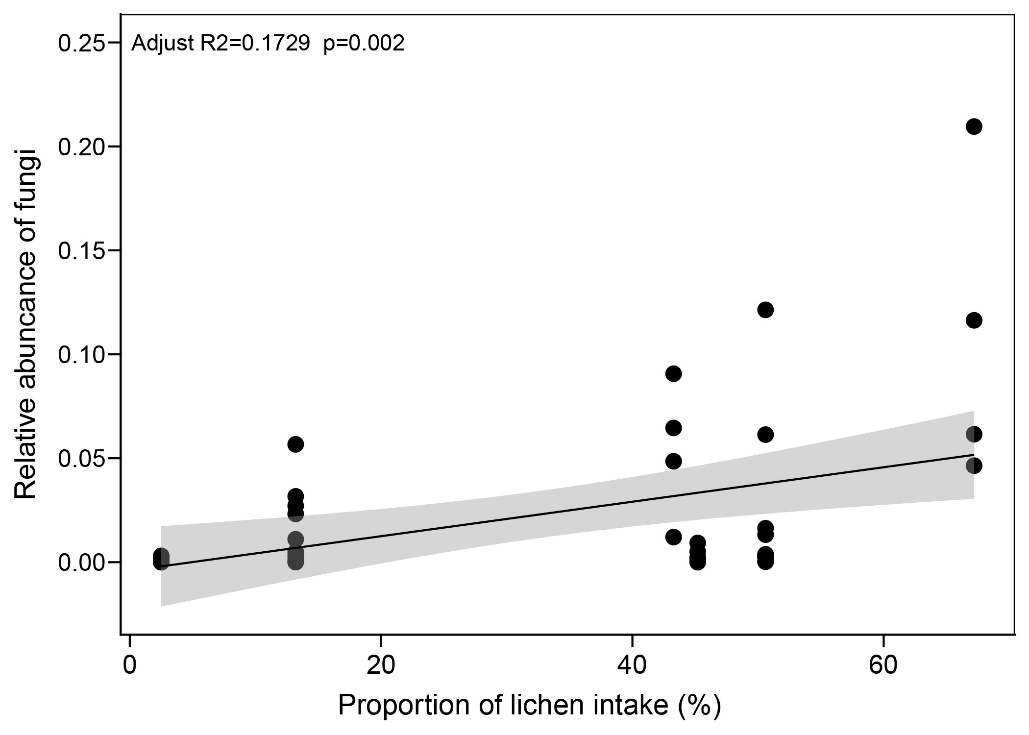
**

**Fig S5**. Correlation between the proportion of lichen intake in diet of three snub-nosed monkeys in our sampling sites (*R. roxellana*: Shennongjia, Mianyang; *R. bieti*: Mt. Lasha, Mangkang, Tacheng; *R.strykeri*: Mt. Gaoligong) and relative abundance of fungi family (Parmeliaceae, Ramalinaceae). Data are based on (this study, Tie J, 2013; Li et al., 2011; Huang et al., 2012; Xiang et al., 2011; Grueter et al., 2009).
